# PDGFC secreted by cancer-associated fibroblasts promotes epithelial-mesenchymal transition and immunosuppression in lung adenocarcinoma: PDGFC CAFs promotes EMT and immunosuppression in LUAD

**DOI:** 10.3724/abbs.2025042

**Published:** 2025-06-11

**Authors:** Meimei Cui, Xiaodi Ding, Yu Jiang, Liying Zhang, Wangkai Cao, Yongming Wang, Zhimei Sheng, Wei Sun, Ai Guo, Lihui Gu, Xiurong Zhang, Wanli Duan, Lihong Shi, Baogang Zhang

**Affiliations:** 1 Department of Clinical Pathology School of Basic Medicine Shandong Second Medical University Weifang 261053 China; 2 Department of Pathology Shaoxing People’s Hospital Shaoxing 312000 China; 3 School of Clinical Medicine Shandong Second Medical University Weifang 261053 China; 4 Department of Thoracic Surgery Translational Medical Center Weifang Second People′s Hospital (Weifang Respiratory Disease Hospital) Weifang 261041 China; 5 Affiliated Hospital of Shandong Second Medical University Weifang 261041 China; 6 Key Laboratory of Molecular Pharmacology and Translational Medicine Shandong Second Medical University Weifang 261053 China; 7 Medical Research Center Shaoxing People’s Hospital Shaoxing 312000 China; 8 School of Rehabilitation Medicine Shandong Second Medical University Weifang 261053 China; 9 School of Basic Medicine Shandong Second Medical University Weifang 261053 China

**Keywords:** cancer-associated fibroblast, platelet-derived growth factor C, lung adenocarcinoma, epithelial-mesenchymal transition, immunosuppression

## Abstract

This study elucidates the mechanisms by which cancer-associated fibroblast (CAF)-derived platelet-derived growth factor C (PDGFC) promotes the progression of lung adenocarcinoma (LUAD) and explores the impact of PDGFC on immune regulation within the tumor microenvironment (TME). Our results show that there is higher expression of PDGFC in CAFs than in nontumor tissue fibroblasts (NFs) and that higher expression of PDGFC is correlated with poor prognosis in LUAD patients. Furthermore, CAF-derived PDGFC promotes epithelial-mesenchymal transition (EMT) in cancer cells as well as matrix metalloproteinase 2 (MMP2) expression through the PDGF receptor A (PDGFRA)-mitogen-activated protein kinase/extracellular signal-regulated kinase (MAPK/ERK) pathway. Moreover, our study demonstrates that CAF-derived PDGFC is essential for the activation and infiltration of fibroblasts in the TME, as well as the inflammatory infiltration of different immune cell types and the immunosuppressive conditions within the TME. In particular, PDGFC induces increased PDGFRA expression in both tumor cells and fibroblasts, which can lead to reciprocally positive feedback to accelerate malignant tumor progression. This discovery provides a novel TME-targeted strategy for LUAD treatment.

## Introduction

Lung adenocarcinoma (LUAD) is a major subtype of lung cancer. Despite advancements in comprehensive treatments, the 5-year survival rate of late-stage LUAD patients remains less than 50% [ 1– 3] . Cancer-associated fibroblasts (CAFs) are located in the tumor microenvironment (TME) and play pivotal roles in TME remodeling, which can promote the development of various cancers, including LUAD [ 4– 10] . CAFs have diverse origins and thus exhibit various heterogeneities of spatial distributions and biological functions [4].

Platelet-derived growth factors (PDGFs) and their receptors are associated with oncogenesis and drug resistance [11]. PDGFC, in particular, is a key member of the PDGF family [12]. PDGFC is widely expressed in the cardiovascular system and has been identified as an effective mitogen [13] that is involved in tissue remodeling, embryonic development [ 14, 15] and angiogenesis [ 16, 17] by interacting with PDGF receptor A (PDGFRA) [ 13, 18] . In addition, PDGFC is expressed in CAFs, and CAF-derived PDGFC has been shown to be related to growth, metastasis, epithelial-mesenchymal transition (EMT) and drug resistance in gastrointestinal and basal-like breast tumors [ 19, 20] . However, the roles and underlying mechanisms of CAF-derived PDGFC in the progression of LUAD remain unclear.

In the present study, we found that there is higher expression of PDGFC in CAFs than in nontumor tissue fibroblasts (NFs) and that increased expression of PDGFC is correlated with poor prognosis in patients with LUAD. Moreover, CAF-derived PDGFC can facilitate the EMT of cancer cells and increase matrix metalloproteinase 2 (MMP2) expression through the PDGFRA-mitogen-activated protein kinase/extracellular signal-regulated kinase (MAPK/ERK) pathway. In addition, CAF-derived PDGFC can induce NF-CAF transformation and the accumulation of suppressive immune cells, including M2 macrophages, in the TME.

## Materials and Methods

### Cells and reagents

The A549 (SCC-110411) and NCI-H1299 (CL-0165) human LUAD cell lines were sourced from Solarbio (Beijing, China) and Pricella (Shijiazhuang, China), respectively. These cell lines were maintained in RPMI medium 1640 (31800; Solarbio) and RPMI-1640 (PM150110; Pricella), both supplemented with 10% fetal bovine serum (FBS). The human macrophage-like THP-1 cell line was acquired from Shanghai Zhong Qiao Xin Zhou Biotechnology Co., Ltd. (Shanghai, China). The cells were cultured in RPMI 1640 medium (Gibco, Carlsbad, USA) supplemented with 20% FBS and 1% penicillin-streptomycin (Solarbio). All the cultures were maintained at 37°C in a humidified atmosphere with 5% CO _2_.

The fibroblast activation protein (FAP) (E1V9V) rabbit mAb (#66562) was purchased from Cell Signaling Technology (Beverley, USA). The PDGF receptor alpha/PDGFRA antibody (sc-398206) and FAP antibody (sc-65398) were obtained from Santa Cruz Biotechnology (Santa Cruz, USA). The following antibodies were procured from Proteintech (Wuhan, China): PDGFC polyclonal antibody (55076-1-AP), E-cadherin polyclonal antibody (20874-1-AP), Vimentin polyclonal antibody (10366-1-AP), MMP2 polyclonal antibody (10373-2-AP), ERK1/2 polyclonal antibody (11257-1-AP), phospho-ERK1/2 (Thr202/Tyr204) polyclonal antibody (28733-1-AP), Alpha tubulin polyclonal antibody, GAPDH (60004-1-Ig), CD80/B7-1 monoclonal antibody (66406-1-Ig), CD206 polyclonal antibody (18704-1-AP), HRP-conjugated affinipure goat anti-mouse IgG (H+L) (SA00001-1), HRP-conjugated affiniPure goat anti-rabbit IgG (H+L) (SA00001-2), and CoraLite488-conjugated goat anti-mouse IgG (H+L) (SA00013-1). A mouse two-step assay kit (PV-6002) and a rabbit two-step assay kit (PV-6002) were obtained from ORIGENE (Beijing, China). The PDGFC polyclonal antibody (bs-5775R) was obtained from Bioss (Beijing, China). The alpha-smooth muscle actin (α-SMA) antibody (#BF9212) was obtained from Affinity Biosciences (Nanjing, China). Goat anti-rabbit IgG H&L (Alexa Fluor® 594) (ab150080) was obtained from Abcam (Cambridge, USA). The additional reagents used in this study included recombinant human PDGFCC (rhPDGFCC, 20 nM; (MedChemExpress, Monmouth Junction, USA), SCH772984 (5 μM; Selleckchem, Houston, USA) and PDGFRA kinase inhibitor 1 (0.2 μM; MedChemExpress).

### Primary CAF and NF isolation

CAFs and NFs were obtained from freshly resected LUAD tissues and matched nontumor lung tissues, respectively, via the tissue block adherence method [ 21, 22] . The cells emerged from the bottom of the tissue blocks after 5–7 days, and an electron microscope (FEI Tecnai G2 Spirit; Thermo Fisher Scientific, Waltham, USA) was used to observe the cell morphology. Western blot analysis was used to detect the expressions of FAP and α-SMA in CAFs and NFs. Both CAFs and NFs were cultured for no more than 10 passages for subsequent experiments.

### Trypan blue staining assay

The cells were prepared and stained with Trypan Blue staining solution obtained from RWD (Shenzhen, China). Then, within three minutes, 10 μL of the cell suspension was pipetted onto the counting chamber. After that, the RWD automatic cell counter was used to count the number of cells, with live cells circled in green and dead cells circled in red. The counted cells were used to evaluate cell viability according to the following formula: cell viability (%) = (total cells – dead blue cells) / total cells × 100%.

### Western blot analysis

Cell lysates were prepared via RIPA lysis buffer (Beyotime, Shanghai, China) supplemented with phenylmethylsulfonyl fluoride (PMSF; Solarbio). Proteins were separated by SDS-PAGE and transferred onto PVDF membranes (Merck Millipore, Molsheim, Germany). After 1 h of blocking in 5% skim milk (Solarbio), the membranes were incubated with primary antibodies overnight at 4°C. Following three washes with TBST (Solarbio), the membranes were treated with HRP-conjugated secondary antibodies (1:8000) at room temperature for 1 h. Protein signals were detected via ECL Western Blotting Substrate (Solarbio) and visualized with the Amersham ImageQuant 800 system (Cytiva; Amersham, Buckinghamshire, UK). The results were quantified via ImageJ software (NIH, Bethesda, USA). Each experiment was repeated three times.

### Enzyme-linked immunosorbent assay (ELISA)

A human PDGFC ELISA kit (JL23863; Shanghai Future Industrial Co., Ltd, Shanghai, China) was used to determine the concentration of PDGFC in the culture media of MRC5 cells, NFs and CAFs. The assay steps included sample addition, biotinylated antibody application, plate washing, addition of enzyme conjugate working solution, further plate washing, substrate application, and addition of the termination solution. The optical density (OD) of each well was measured at 450 nm. The sample concentration was determined via a standard curve. Each experiment was repeated three times.

### Transwell assay

The migratory ability of LUAD cells was assessed via Transwell inserts (Corning, New York, USA). A total of 1 × 10 ^6^ LUAD cells suspended in 200 μL of serum-free medium were seeded into the upper chamber, while 500 μL of medium containing NF-conditioned media (NF-CM), CAF-CM or rhPDGFCC was placed in the lower chamber. After 18 h of incubation at 37°C, non-migrated cells on the upper surface of the membrane were gently removed with a cotton swab. The migrated cells on the lower surface were fixed with methanol and stained with Giemsa Stain Solution (Solarbio). Migrated cells were counted in eight randomly chosen fields under an electron microscope (FEI Tecnai G2 Spirit; Thermo Fisher Scientific). Each experiment was repeated three times.

### Wound healing assay

For the wound healing experiment, we used a 10-μL pipette tip to vertically scratch the cells in a 6-well plate. LUAD cells were starved in serum-free culture medium for 24 h to eliminate the influence of cell proliferation. The cells were treated with different cell supernatants, and then, images of the wound area were captured at 0 h and 24 h under a microscope. Representative boundaries of the wound were drawn via straight lines. Each experiment was repeated three times.

### Immunohistochemistry (IHC)

The tissues were fixed in 10% formaldehyde, embedded in paraffin and sectioned into 4-μm slices. The sections were deparaffinized, rehydrated, and subjected to antigen retrieval via All-purpose Powerful Antigen Retrieval Solution (Beyotime). Then, the sections were incubated with 3.0% H _2_O _2_ to eliminate the activity of endogenous peroxidase and then with normal goat serum to block nonspecific binding sites. Next, the sections were incubated with primary antibodies overnight at 4°C. After incubation with secondary antibodies, the sections were stained with a DAB substrate kit (Beyotime) and counterstained with Mayer’s hematoxylin (Solarbio). After dehydration and sealing, the sections were observed and evaluated by two pathologists independently. Each experiment was repeated at least three times.

### Immunofluorometric assay

For the tissue samples, the paraffin-embedded sections were subjected to antigen retrieval by heating in All-purpose Powerful Antigen Retrieval Solution (Beyotime). Endogenous peroxidase activity was quenched with 3% hydrogen peroxide in methanol. For the cell samples, the cells were fixed on coverslips with 4% formalin for 15 min. To minimize nonspecific binding, the samples were blocked at room temperature for 30 minutes via a fluorescent blocking solution containing 10% goat serum and 0.3% Triton X-100. The samples were incubated with primary antibodies overnight at 4°C, followed by incubation with fluorochrome-conjugated secondary antibodies for 30 min at room temperature. Finally, DAPI (Solarbio) was used to highlight the cell nuclei. Each experiment was repeated three times.

### Hematoxylin and eosin (H&E) staining

Extracted tissues were fixed in 4% paraformaldehyde, and 3-μm-thick sections were cut from paraffin-embedded blocks. After deparaffinization, the sections were stained with hematoxylin to color the nuclei and with eosin to color the cytoplasm. The prepared slides were examined under a microscope, and images were captured for further analysis.

### Bioinformatics analysis

The University of Alabama at the Birmingham Cancer Data Analysis Portal (UALCAN) ( https://ualcan.path.uab.edu/index.html) is an interactive platform for analyzing cancer OMICS data. The Human Protein Atlas (HPA) ( https://www.proteinatlas.org/) was used to assess PDGFC expression in normal and LUAD human lung tissues. The Kaplan-Meier plotter ( https://kmplot.com/analysis/) was used to evaluate the relationship between gene expressions (mRNA, miRNA, protein, and DNA) and survival across 35,000+ samples from 21 cancer types. GEPIA2 ( http://gepia2.cancer-pku.cn/#index) was used to analyze the correlations between PDGFRA and MMP2. LinkedOmics ( https://www.linkedomics.org/login.php) offers multiomics data from 32 TCGA cancer types and 10 Clinical Proteomics Tumor Analysis Consortium (CPTAC) cancer cohorts. Gene Set Cancer Analysis (GSCA) ( https://guolab.wchscu.cn/GSCA/#/) is a platform for multigroup analysis that visualizes potential signaling pathways involved in gene groups across solid tumors on the basis of TCGA data [23].

TIMER ( www.cistrome.shinyapps.io/timer/) is a gene immune analysis platform that enables direct immune analysis of specific genes in certain cancers and visualizes the results for researchers’ use. Through the ″gene module″, we conducted a correlation analysis of PDGFC with immune infiltration levels in LUAD. Owing to the differences between different algorithms, this study prioritized the EPIC and quantTIseq algorithms [24]. The TISIDB ( http://cis.hku.hk/TISIDB/) utilizes high-throughput genetic data to identify and predict associations between specific genes and tumor immune cell infiltration. To further investigate the role of PDGFC in regulating immune infiltration in LUAD, we used TISIDB to explore the correlation between PDGFC expression and immunoinhibitors. A *P* value of less than 0.05 was considered to indicate statistical significance.

We explored the downstream pathways of PDGFC through Kyoto Encyclopedia of Genes and Genomes (KEGG; https://www.kegg.jp/) pathway analysis. Additionally, we performed a survival analysis to investigate the relationship between MMP2 and LUAD patient survival via the Kaplan-Meier Plotter ( https://kmplot.com/analysis/) website.

### Animal experiments

Animal experiments were performed with the approval of the Ethics Committee at Shandong Second Medical University. A total of 2 × 10 ^6^  A549 cells alone or a mixture of 2 × 10 ^6^  A549 cells combined with 5 × 10 ^5^ NFs or 5 × 10 ^5^ CAFs (4:1 ratio) were suspended in 100 μL of phosphate-buffered saline (PBS) and subcutaneously injected into 4–6-week-old female nude mice (BALB/c-nu; Jinan Pengyue Experimental Animal Breeding Co., Ltd., Jinan, China). The tumor volume and weight were recorded every 2 days, and the tumor volume was calculated via the following formula: tumor volume = length × width²/2. When the tumor volume reached 60–80 mm³, rhPDGFCC (10 ng/kg) was injected intraperitoneally every 7 days for 5 total doses. At the end of the study, the animals were euthanized, the primary tumors were excised, and metastasis was evaluated. The tissue samples were fixed in 10% buffered formalin for subsequent IHC analysis.

### Statistical analysis

All experiments were independently repeated three times, and the data are presented as the mean ± SD to account for experimental variability. Statistical analysis and data visualization were performed via GraphPad Prism software version 9.5.1 (San Diego, USA). To assess the significance of the observed differences, a two-tailed unpaired Student’s *t* test and one-way analysis of variance (ANOVA) with Tukey’s multiple comparison test were used. *P*  < 0.05 was considered to indicate statistical significance.

## Results

### PDGFC is associated with poor prognosis in LUAD patients

**Figure 1:**
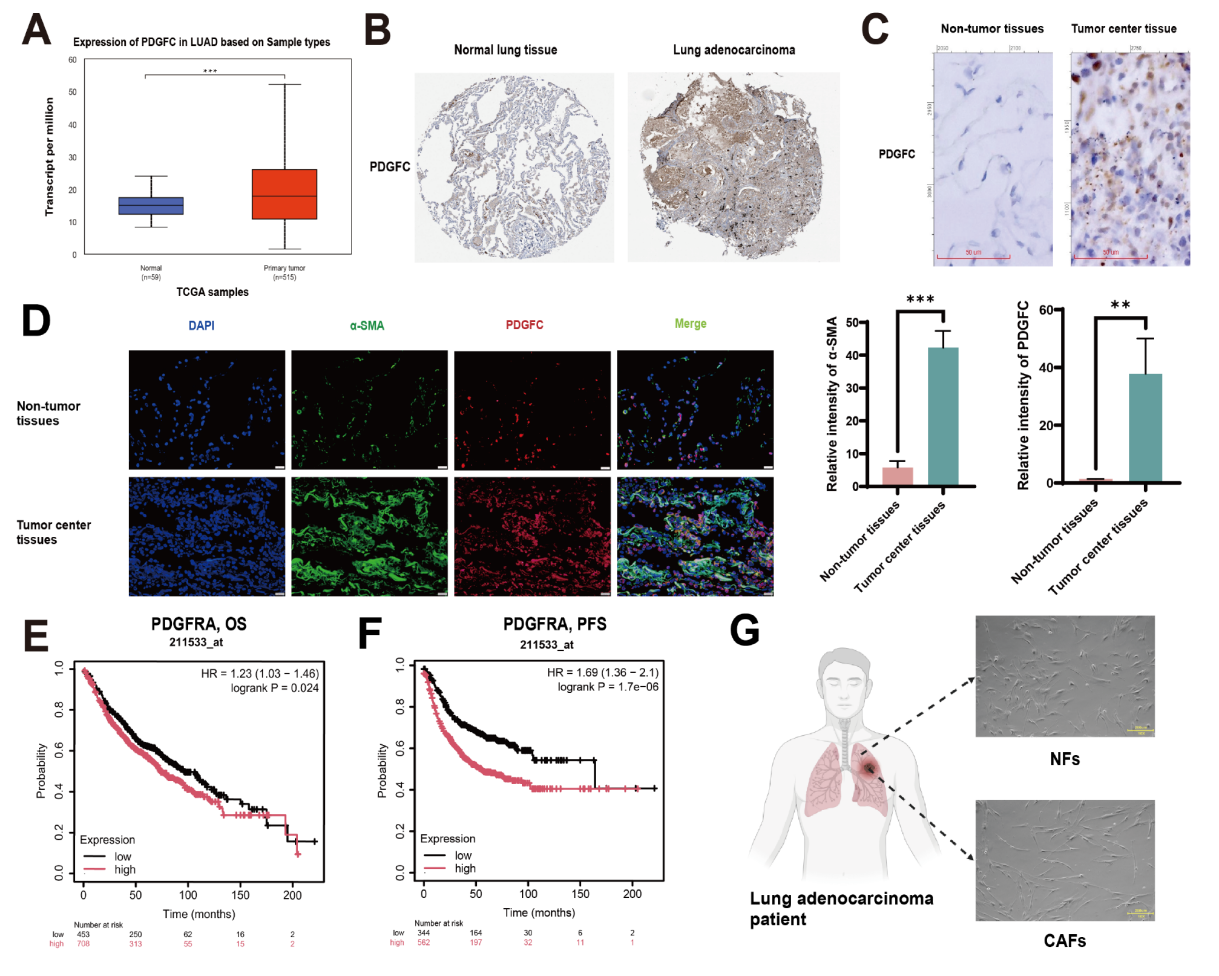
Figure 1 PDGFC is associated with poor prognosis in LUAD patients (A) The expression of PDGFC in normal human lung tissues and LUAD tissues was analyzed via the UALCAN database. (B) PDGFC expression in normal human lung tissues and LUAD tissues analyzed via the HPA database. Scale bar: 200 µm. (C) PDGFC expression in nontumor and tumor center tissues from LUAD patients was detected by IHC. Scale bar: 20 µm. (D) Co-localization of PDGFC and α-SMA detected by dual immunofluorescence staining. Scale bar: 20 µm. (E) The relationship between PDGFRA and overall survival (OS) in LUAD patients was analyzed by Kaplan-Meier plotter database. (F) The relationship between PDGFRA and progression-free survival (PFS) in LUAD patients was analyzed by Kaplan Meier plotter database. (G) Representative microscopy images of CAFs and paired NFs isolated from the center and nontumor tissues of LUAD patients. Scale bar: 50 µm. Data are expressed as the mean ± SD, n ≥ 3. **P < 0.01, ***P < 0.001.

Both the UALCAN database ( Figure 1A) and the HPA database ( Figure 1B) were used to explore the roles of PDGFC in the progression of LUAD. The data revealed that PDGFC expression was elevated in LUAD tissues compared with nontumor tissues, and this finding was further confirmed by our IHC analysis ( Figure 1C). Notably, the results of the IHC analysis also indicated that PDGFC was primarily localized in the tumor stroma ( Figure 1B). Further dual immunofluorescence analysis revealed the colocalization of PDGFC and α-SMA, suggesting relatively increased expression of PDGFC in CAFs ( Figure 1D). Furthermore, the Kaplan-Meier plotter database revealed that high expression of a specific receptor of PDGFC, PDGFRA, was negatively correlated with overall survival (OS) and progression-free survival (PFS) in LUAD patients ( Figure 1E,F). These results indicate that high expression levels of PDGFC and PDGFRA are associated with an unfavorable prognosis in patients with LUAD.

**Figure 2:**
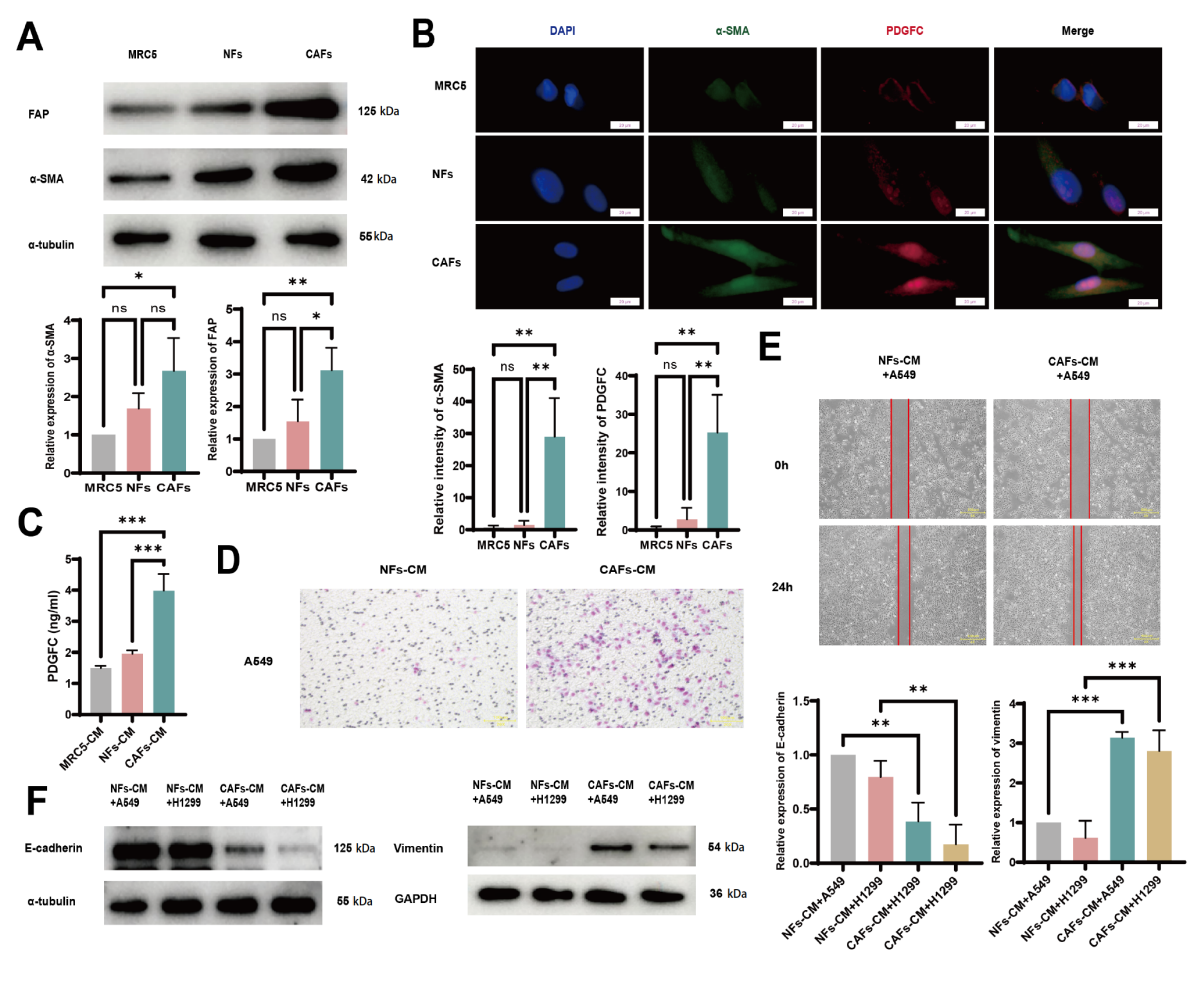
Figure 2 CAFs promote LUAD invasion, migration, and EMT (A) The expressions of CAF markers (FAP and α-SMA) in MRC5 cells, NFs and CAFs were determined by western blot analysis. (B) Co-expressions of α-SMA and PDGFC in MRC5 cells, NFs and CAFs were detected by dual immunofluorescence staining. Scale bar: 20 µm. (C) Concentrations of PDGFC in the supernatants of MRC5 cells, NFs and CAFs detected by ELISA. (D) Invasive ability of A549 cells treated with NF-CM or CAF-CM, as detected by transwell assay. Scale bar: 50 µm. (E) The migratory ability of A549 cells co-cultured with NFs or CAFs was detected by wound healing assay. Scale bar: 400 µm. (F) E-cadherin and vimentin expressions in A549 and H1299 cells treated with NF-CM or CAF-CM for 24 h were detected by western blot analysis. Data are expressed as the mean ± SD, n ≥ 3. *P < 0.05, **P < 0.01, ***P < 0.001.

Primary CAFs and paired NFs were subsequently obtained from LUAD tumor tissues and nontumor lung tissues, respectively. NFs were relatively short and thick with smaller cell sizes and relatively regular shapes, whereas CAFs exhibited elongated shapes, relatively larger sizes, irregular shapes and more obvious extensibility ( Figure 1G). Western blot analysis revealed increased expressions of α-SMA and FAP in CAFs compared with those in MRC5 cells and NFs ( Figure 2A). In accordance with the colocalization of PDGFC and α-SMA in LUAD tissues, dual immunofluorescence detection also revealed the co-expression of α-SMA and PDGFC in the majority of CAFs ( Figure 2B). Moreover, ELISA analysis revealed higher PDGFC expression in CAF-CM than in MRC5-CM and NF-CM ( Figure 2C).

### CAF-derived PDGFC induces EMT in LUAD cells via the PDGFRA-MAPK-ERK signaling pathway

Transwell and wound healing assays showed that CAF-CM significantly increased the migratory and invasive abilities of both A549 and H1299 cells ( Figure 2D,E and Supplementary Figure S1A,B). Since EMT can result in increased migration and invasion of tumor cells, we wondered whether CAF-CM could induce EMT in LUAD cells. Western blot analysis revealed that CAF-CM downregulated the expression of the epithelial marker E-cadherin but upregulated the expression of the mesenchymal marker vimentin ( Figure 2F), indicating that CAF-CM facilitates the EMT of LUAD cells.

**Figure 3:**
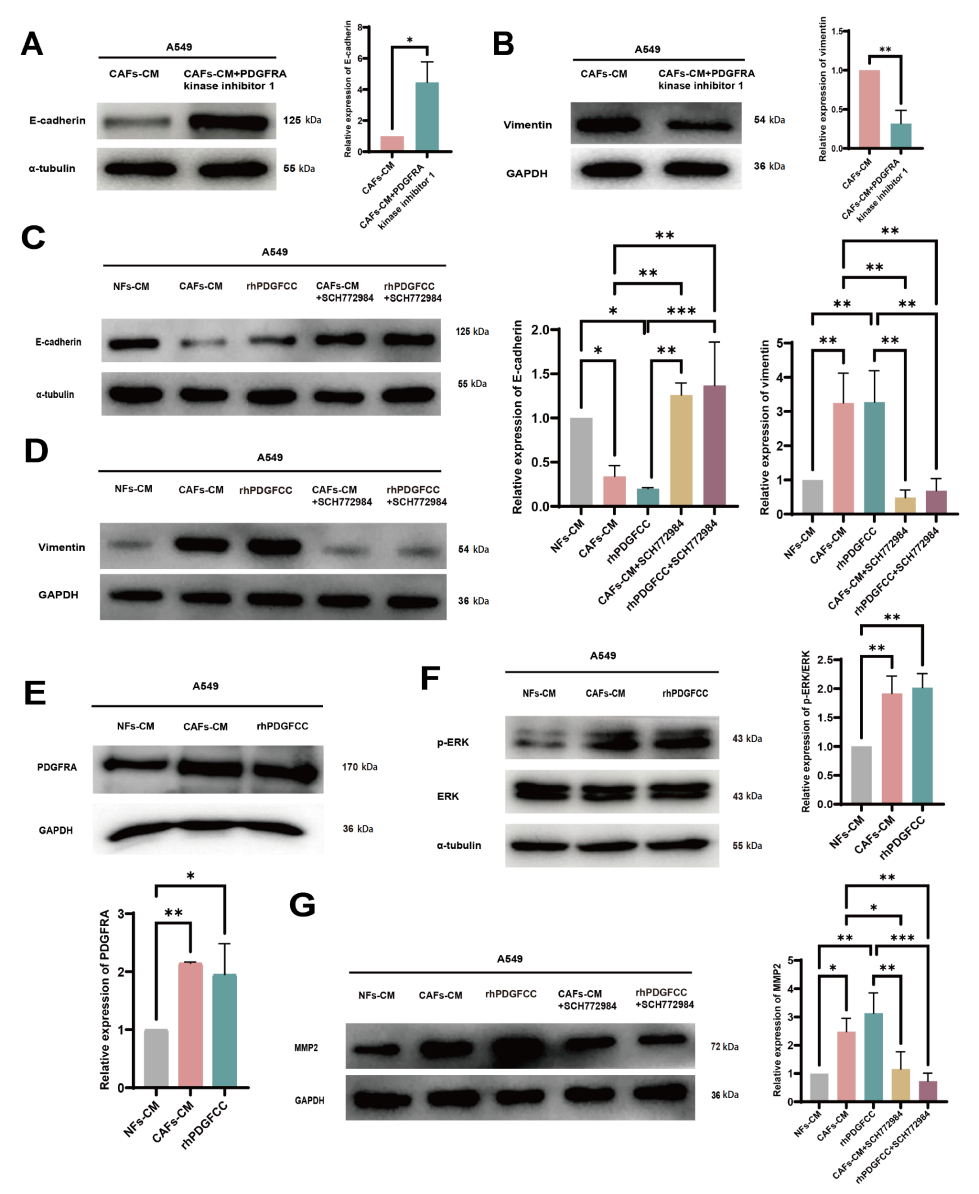
Figure 3 CAF-derived PDGFC induces EMT in LUAD cells via the PDGFRA-MAPK-ERK signaling pathway (A) Western blot analysis of E-cadherin expression in A549 cells treated with CAF-CM with or without PDGFRA kinase inhibitor 1. (B) Western blot analysis of vimentin expression in A549 cells treated with CAF-CM with or without PDGFRA kinase inhibitor 1. (C) Western blot analysis of E-cadherin expression in A549 cells treated with NF-CM, CAF-CM, rhPDGFCC, CAF-CM + SCH77298 or rhPDGFCC + SCH77298. (D) Western blot analysis of vimentin expression in A549 cells treated with NF-CM, CAF-CM, rhPDGFCC, CAF-CM + SCH77298 or rhPDGFCC + SCH77298. (E) Western blot analysis of PDGFRA expression in A549 cells treated with NF-CM, CAF-CM or rhPDGFCC. (F) Western blot analysis of p-ERK and ERK in A549 cells treated with NF-CM, CAF-CM or rhPDGFCC. (G) Western blot analysis of MMP2 expression in A549 cells treated with NF-CM, CAF-CM, rhPDGFCC, CAF-CM + SCH77298 or rhPDGFCC+SCH77298. Data are expressed as the mean ± SD, n ≥ 3. *P < 0.05, **P < 0.01, ***P < 0.001.

Next, to determine whether CAF-derived PDGFC plays a role in inducing EMT in LUAD cells, we treated LUAD cells with CAF-CM or CAF-CM + PDGFRA kinase inhibitor 1. Western blot analysis revealed that CAF-CM-induced EMT in A549 cells was markedly inhibited by PDGFRA kinase inhibitor 1 ( Figure 3A,B). Moreover, rhPDGFCC alone was added to the culture medium of LUAD cells, and as expected, rhPDGFCC caused EMT in A549 and H1299 cells, similar to CAF-CM ( Figure 3C,D and Supplementary Figure S1C,D). Thus, CAF-derived PDGFC likely promoted the EMT of LUAD cells. Furthermore, western blot analysis demonstrated a marked increase in PDGFRA expression in A549 cells co-cultured with CAF-CM or rhPDGFCC ( Figure 3E), suggesting that CAF-derived PDGFC can promote PDGFRA expression in LUAD cells, which results in continuous reciprocal feedback between the PDGFC-PDGFRA interaction.

To explore the mechanism underlying PDGFC-induced EMT in LUAD cells, KEGG pathway enrichment analysis was conducted via the LinkedOmics database. The results revealed that PDGFC-related genes are enriched mainly in the MAPK and phosphatidylinositol 3-kinase/protein kinase B (PI3K-Akt) signaling pathways in cancer ( Supplementary Figure S2A). GSCA revealed that PI3K/Akt signaling was suppressed in LUAD ( Supplementary Figure S2B), so we assessed the function of MAPK signaling in LUAD cells cultured with CAF-CM or rhPDGFCC. Western blot analysis demonstrated that both CAF-CM and rhPDGFCC significantly increased p-ERK1/2 expression ( Figure 3F and Supplementary Figure S2C), whereas the ERK pathway inhibitor SCH772984 attenuated both CAF-CM- and rhPDGFCC-induced EMT in A549 and H1299 cells ( Figure 3C,D).

MMP2 is correlated with poor prognosis in cancers, and the ERK pathway can regulate MMP2 expression. Consistently, our Kaplan-Meier plotter database analysis revealed a negative correlation between MMP2 expression and OS/PFS in LUAD patients ( Supplementary Figure S2D,E). Additionally, GEPIA 2 database analysis revealed a positive correlation between PDGFRA and MMP2 ( Supplementary Figure S2F). To further validate the relationship between PDGFC and MMP2, CAF-CM or rhPDGFCC was added to the culture medium of A549 cells. Interestingly, both CAF-CM and rhPDGFCC significantly promoted MMP2 expression, whereas the increase in MMP2 expression was markedly inhibited by the ERK inhibitor SCH772984 ( Figure 3G). These results are in accordance with the findings in the UALCAN database, which revealed higher MMP2 expression in LUAD tissues than in matched normal lung tissues ( Supplementary Figure S2G), indicating that CAF-derived PDGFC could upregulate MMP2 expression through the modulation of the MAPK-ERK signaling pathway.

### CAF-derived PDGFC promotes CAF accumulation and immune suppression in the TME

**Figure 4:**
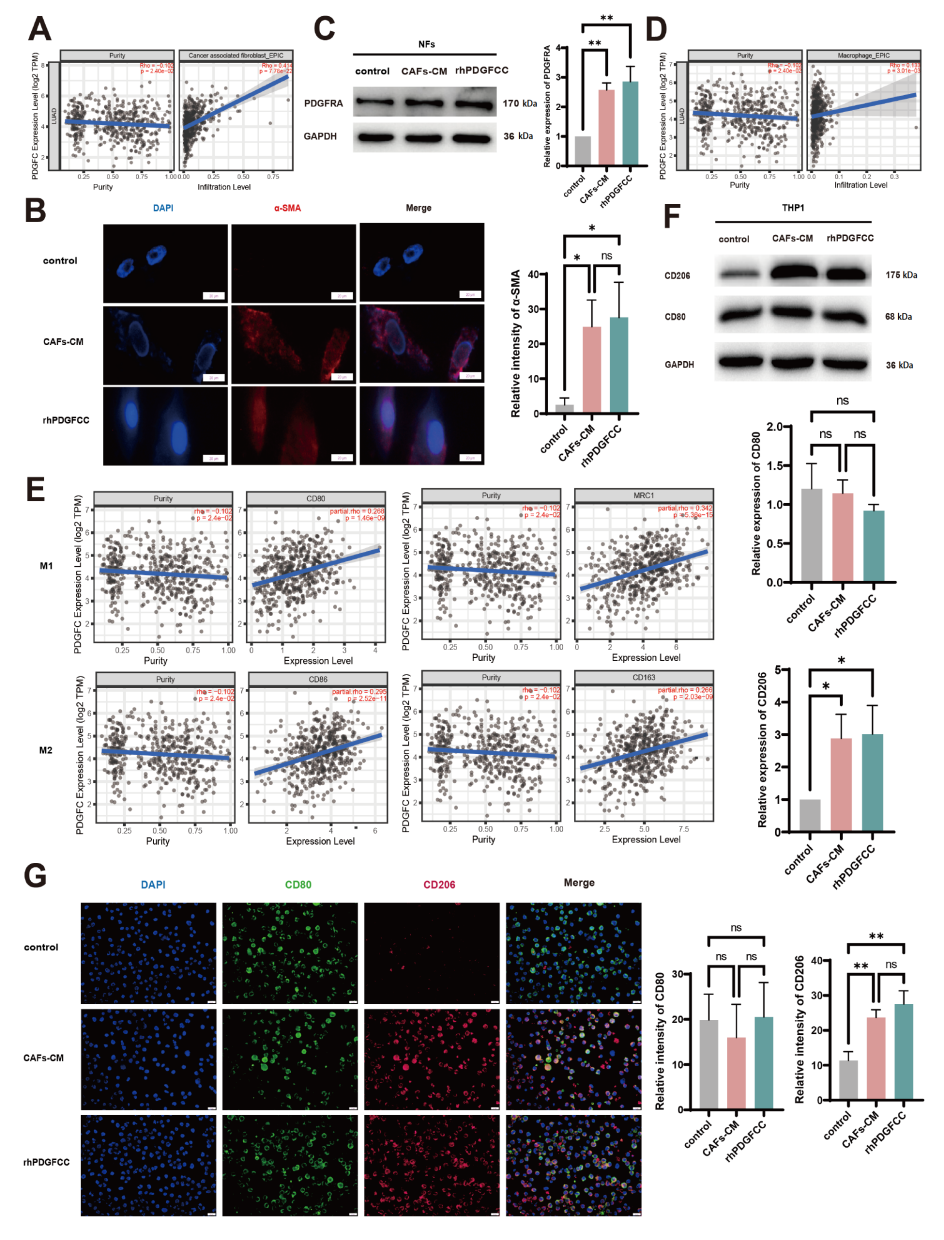
Figure 4 CAF-derived PDGFC promotes CAF accumulation and immune suppression in the TME (A) The correlation of PDGFC expression with CAF accumulation in LUAD. (B) Immunofluorescence staining was used to detect the expression of α-SMA. Scale bar: 20 µm. (C) PDGFRA expression in NFs, CAF-CM-treated NFs and rhPDGFCC-treated NFs was detected by western blot analysis. (D) The correlation of PDGFC expression with macrophage infiltration in LUAD. (E) Correlation of PDGFC expression with M1/M2 markers in LUAD. (F) Western blot analysis of CAF-CM/rhPDGFCC-induced expressions of M1 and M2 markers in THP-1 cells. (G) Immunofluorescence staining of CAF-CM/rhPDGFCC-induced expressions of CD206 and CD80 in THP-1 cells. Scale bar: 20 µm. Data are expressed as the mean ± SD, n ≥ 3. *P < 0.05, **P < 0.01.

In the malignant progression of LUAD, the evolution of various constituents in the TME should also be considered. To further explore the roles of CAF-derived PDGFC in the development of LUAD, we analyzed the TIMER 2.0 database and found that PDGFC was positively correlated with the infiltration of CAFs in the TME ( Figure 4A). Subsequently, immunofluorescence detection revealed significantly increased α-SMA expression in the CAF-CM/rhPDGFCC-treated NFs, suggesting that PDGFC could activate NFs and thus induce NF-CAF transformation ( Figure 4B). Moreover, a significant increase in PDGFRA expression in CAF-CM/rhPDGFCC-treated NFs was detected via western blot analysis ( Figure 4C), which further enhanced the PDGFC-PDGFRA effect via positive feedback, leading to the continuous accumulation of CAFs in the TME.

Moreover, TIMER 2.0 database analysis revealed the relationship between PDGFC expression and immune cell infiltration in LUAD tissues. The numbers of infiltrating CD4 ^+^ T cells (Cor = 0.196, *P*  = 1.18 × 10 ^–5^), macrophages (Cor = 0.133, *P*  = 3.01 × 10 ^–3^), neutrophils (Cor = 0.175, *P* = 9.56 × 10 ^–5^), and Tregs (Cor = 0.257, *P*  = 7.03 × 10 ^–9^) were positively correlated with PDGFC expression, whereas the number of natural killer (NK) cells was negatively correlated with PDGFC expression (Cor = –0.124, *P*  = 6.01 × 10 ^–3^) ( Figure 4E and Supplementary Figure S3A–G). Furthermore, biomarkers of both M1 (CD80, CD86 and CD68) and M2 (multiligand endocytic receptor mannose receptor (CD206/MRC1)) macrophages were also positively correlated with PDGFC expression. Although the TIMER 2.0 database revealed no obvious difference between the correlations of M1 and M2 macrophages with PDGFC expression, our western blot analysis and immunofluorescence staining results revealed that the M2 marker CD206 was upregulated in CAF-CM/rhPDGFCC-treated THP-1 cells, whereas the M1 marker CD80 was not, indicating that PDGFC can promote macrophage polarization toward the M2 phenotype ( Figure 4F,G). Consistently, a positive correlation was identified between PDGFC and biomarkers of the N2 state of tumor-associated neutrophils ( Supplementary Figure S3H–J) [25]. Moreover, there were strong correlations between PDGFC expression and several immunosuppressive biomarkers, including transforming growth factor beta 1 (TGFB1), colony-stimulating factor 1 receptor (CFS1R), programmed death 1 ligand-1 (CD274/PD-L1), programmed cell death 1 ligand 2 (PDCD1LG2/PD-L2), vascular endothelial growth factor (VEGF) receptor-2 (Flk1/KDR), interleukin‐10 receptor subunit beta gene (IL10RB) and hepatitis A virus cellular receptor 2 (HAVCR2) in LUAD ( Supplementary Figure S4A). Overall, these data suggested that CAF-derived PDGFC promoted tumor progression through the induction of immune suppression in the TME.

### CAF-derived PDGFC promotes LUAD progression *in vivo*

**Figure 5:**
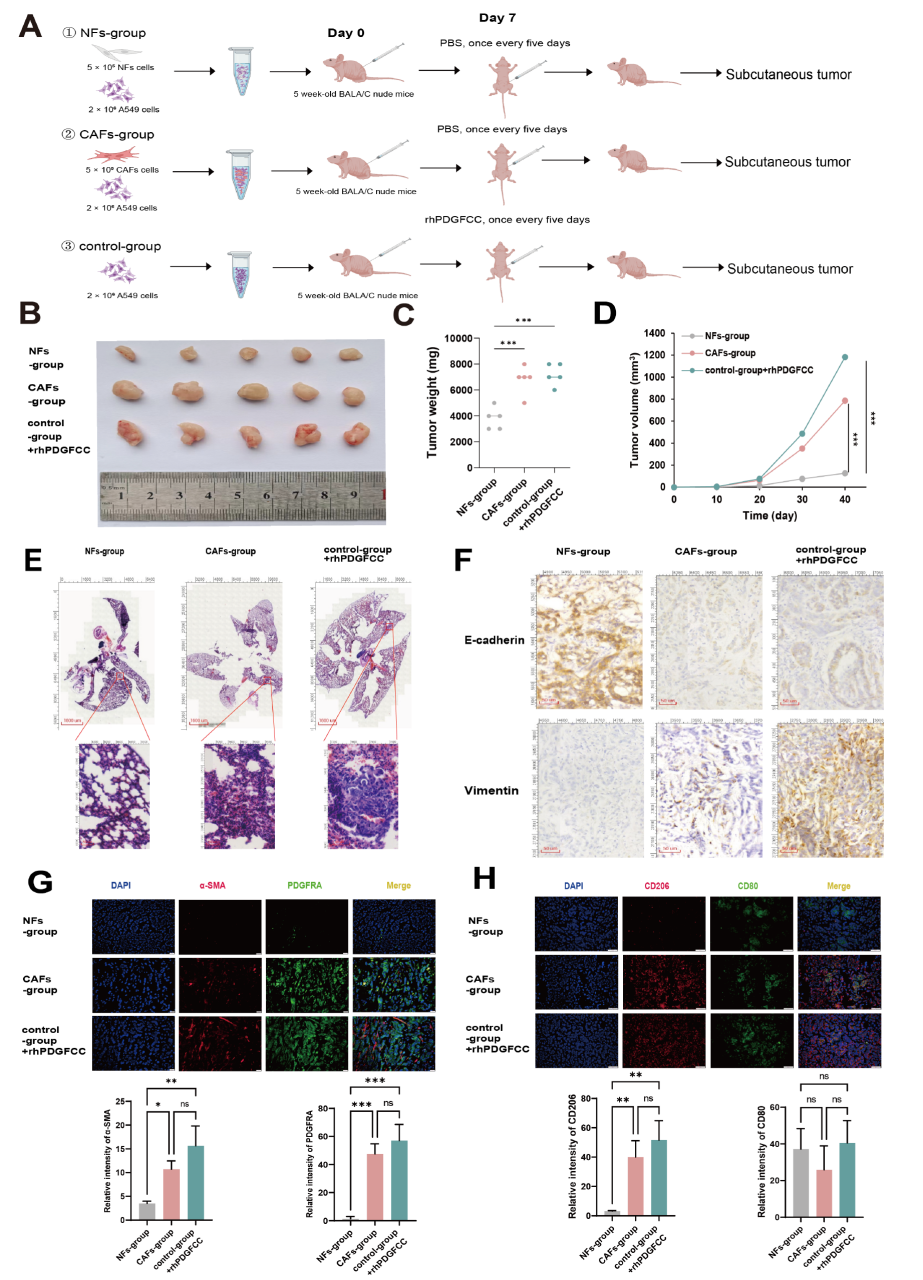
Figure 5 CAF-derived PDGFC promotes LUAD progression *in viv*o (A,B) Three groups of xenograft mouse models were established: the NF group, CAF group and control group. (C,D) The weight (mg) and volume (mm3) of xenografts. (E) H&E-stained histological images of the lungs and corresponding intrapulmonary metastases of the NF group, CAF group and control group + rhPDGFCC. Scale bar: 1600 µm (low magnification) and 50 µm (high magnification). (F) IHC staining of E-cadherin and vimentin in xenograft tissues. Scale bar: 20 µm. (G) Immunofluorescence staining of α-SMA and PDGFRA expressions in xenograft tissues. Scale bar: 20 µm. (H) Immunofluorescence staining of CD206 and CD80 in xenograft tissues. Scale bar: 20 µm. Data are expressed as the mean ± SD, n ≥ 3. *P < 0.05, **P < 0.01, ***P < 0.001.

To validate the function of CAF-derived PDGFC *in vivo*, we established three groups of xenograft mouse models, the control group, the NF group and the CAF group, via the subcutaneous injection of A549 cells, NFs + A549 cells and CAFs + A549 cells, respectively, into BALB/c-nu mice. When the volume of the implanted tumors reached 60–80 mm ^3^, the control group was given rhPDGFCC (10 ng/kg) intraperitoneally weekly for 5 weeks ( Figure 5A). The results showed that CAFs and rhPDGFCC, rather than NFs, markedly promoted tumor growth *in vivo* ( Figure 5B–D). In addition, CAFs and rhPDGFCC increased both the size and number of pulmonary metastatic nodules ( Figure 5E). Consistent with our finding that CAFs promoted the EMT of A549 cells, xenografts of the CAF group and control group + rhPDGFCC showed significantly decreased expression of E-cadherin and increased expression of vimentin ( Figure 5F). Importantly, xenografts of the CAF group and control group + rhPDGFCC presented increased expressions of α-SMA, PDGFRA and the M2 marker CD206 compared with those of the NF group ( Figure 5G,H).

## Discussion

TME-targeted therapy is promising for cancer treatment [26]. CAFs are the most abundant noncancer cells in the TME and can secrete various biological molecules that facilitate tumor initiation and progression [27]. Consequently, targeting CAFs has emerged as a vital strategy. Nonetheless, the precise mechanisms through which CAFs drive tumor progression remain unclear.

This study revealed that CAF-derived PDGFC can induce EMT through the activation of the MAPK/ERK signaling pathway, thereby driving the invasion and metastasis of LUAD. This finding is in accordance with the reported function of PDGFC in other types of tumors [ 20, 28] . In particular, our study revealed that CAF-derived PDGFC significantly increased the expression of the specific receptor of PDGFC, PDGFRA, in LUAD cells. Therefore, the reciprocal feedback interaction between PDGFRA in LUAD cells and CAF-derived PDGFC efficiently promotes EMT.

Additionally, as a receptor tyrosine kinase (RTK), PDGFRA has been reported to be associated with cell proliferation, migration, survival, aggressiveness and poor prognosis of some types of cancers [ 29- 31] . Importantly, our results revealed that CAF-derived PDGFC also increased the expression of PDGFRA in fibroblasts as well as the PDGFC-induced expression of α-SMA in NFs. Accordingly, we deduce that CAF-derived PDGFC can facilitate NF-CAF transformation, increase the secretion of PDGFC and further enhance the PDGFC-PDGFRA interaction, which leads to fibrosis of the TME and the malignant progression of LUAD.

Moreover, consistent with previous reports, our results showed that PDGFC could regulate the expression of MMP2 through the MAPK/ERK pathway [ 32– 35] . By degrading the extracellular matrix, *MMP2* is a PDGFRA-related gene that is associated with poor prognosis in LUAD patients [ 36, 37] , and this finding suggests a potential therapeutic target for LUAD.

Furthermore, our results indicate that PDGFC could facilitate inflammatory infiltration and exert immunosuppressive effects in the TME. The expression level of PDGFC is positively related to TME infiltration of CD4 ^+^ T cells (Treg cells), macrophages and neutrophils, which is consistent with the chronic inflammatory nature of tumors. Even so, PDGFC contributes to the immunosuppressive characteristics of the TME by inducing the infiltration and polarization of Treg cells, M2 macrophages and N2 neutrophils in the TME, while restraining the infiltration of immunocompetent NK cells. Moreover, immunoinhibitors TGFB1, CFS1R, PD-L1, PD-L2, KDR, IL10RB and HAVCR2 may have synergistic effects with PDGFC in modulating immunosuppression. Thus, PDGFC can be used not only as a diagnostic indicator for LUAD but also as a potential immunotherapy target.

Nevertheless, the limitations of this study should be mentioned. In several reported studies, CAFs exhibit pleiotropic effects on tumor progression because of their heterogeneous origins and distributions [ 38– 40] . Unfortunately, our study did not delve into these complexities of CAFs, and more valuable insights into various subsets of CAFs are needed.

In conclusion, this study revealed that CAF-derived PDGFC plays significant roles in the invasion, migration and EMT of LUAD cancer cells. Importantly, PDGFC induces increased PDGFRA expression in both tumor cells and fibroblasts, which leads to reciprocally positive feedback, accelerating the fibrotic TME and malignant progression of tumors. In particular, PDGFC is crucial for immune suppression in the TME, which further promotes tumor progression. This significant study provides a novel TME-targeted therapy for LUAD.

## Supporting information

24728Supplementary_Data_25

## Supplementary Data

Supplementary data is available at *Acta Biochimica et Biophysica Sinica* online.
