# Supplementary material for: PDGFC secreted by cancer-associated fibroblasts promotes epithelial-mesenchymal transition and immunosuppression in lung adenocarcinoma: PDGFC CAFs promotes EMT and immunosuppression in LUAD
Source: Acta Biochim Biophys Sin (Shanghai). 2025 Jun 11;57(10):1625–35. doi: 10.3724/abbs.2025042 (PMC12616728; doi:10.3724/abbs.2025042)
Supplement: 24728Supplementary_Data_25 [file 24728Supplementary_Data_25.3.6.docx]

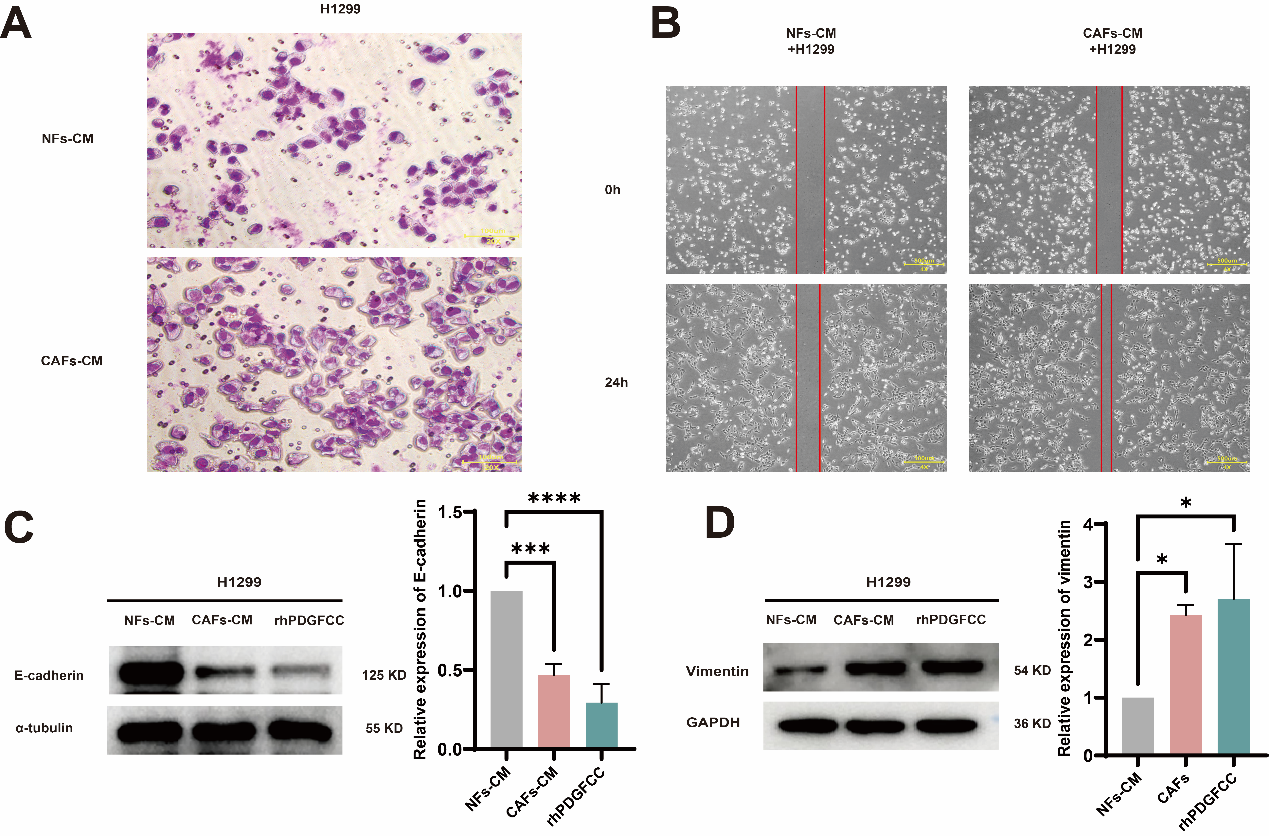
**Supplementary Figure S1. CAFs promote H1299 cell invasion, migration, and EMT** (A) Invasive ability of H1299 cells co-cultured with NFs or CAFs detected by transwell assay. (B) Migratory ability of H1299 cells co-cultured with NFs or CAFs detected by wound healing assay. (C) Western blot analysis of E-cadherin expression in H1299 cells co-cultured with NFs, CAFs, or rhPDGFCC for 24 hours. (B) Western blot analysis of Vimentin expression in H1299 cells co-cultured with NFs, CAFs, or rhPDGFCC for 24 h.


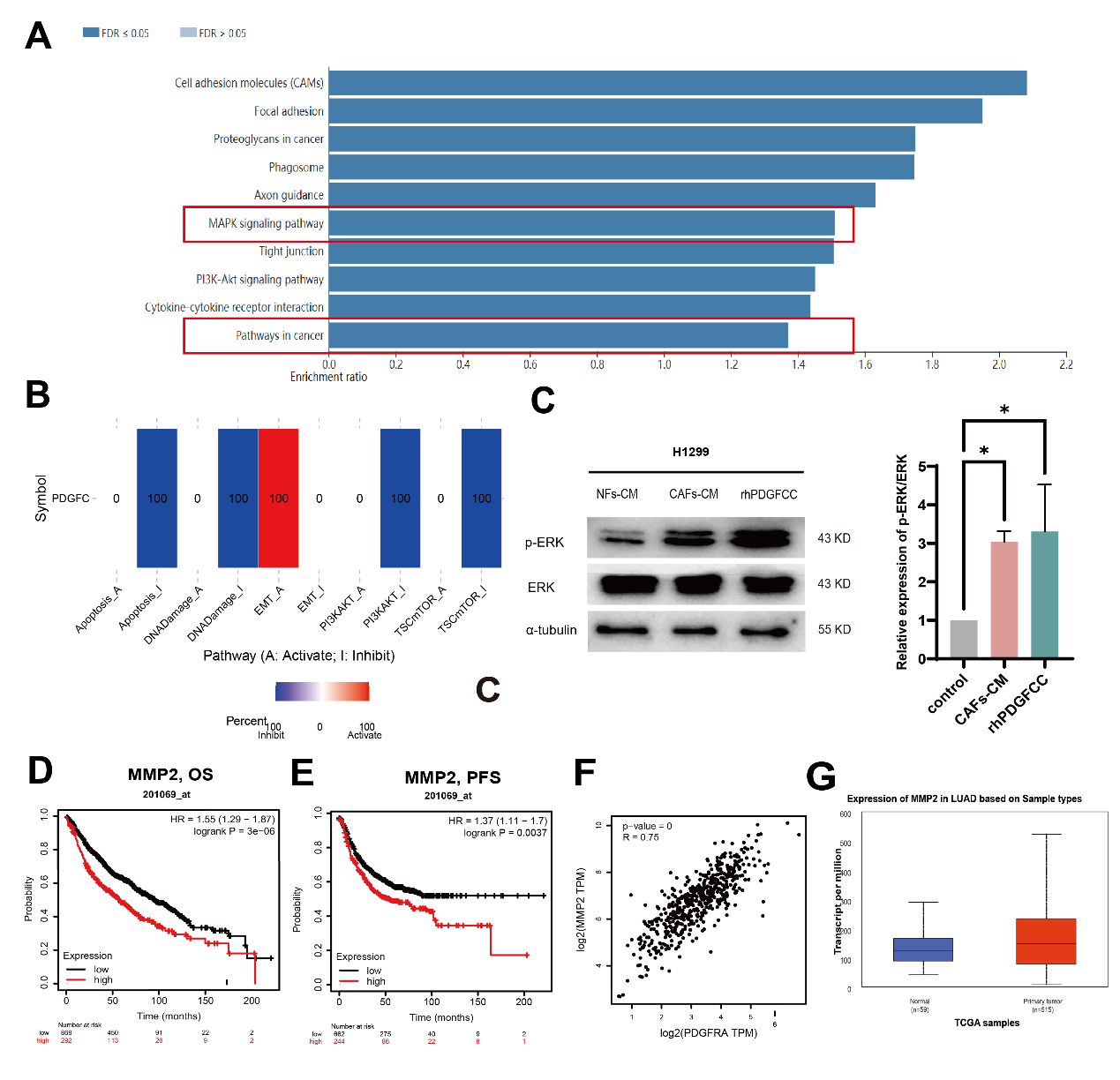


**Supplementary Figure S2. CAFs-derived PDGFC** **activates MAPK/ERK pathway in LUAD** (A) Functional enrichment analysis of PDGFC in LUAD using LinkedOmics database. (B) GSCA database analysis of PDGFC associated pathway in LUAD. (C) Western blot analysis of MAPK/ERK pathway in A549 cells co-cultured with NFs, CAFs, or rhPDGFCC. (D) The Kaplan Meier Plotter database analysis of the relationship between MMP2 and overall survival (OS) in LUAD. (E) The Kaplan Meier Plotter database analysis of the relationship between MMP2 and progression-free survival (PFS) in LUAD. (F) Prediction of the correlation between PDGFRA and MMP2 in LUAD using the GEPIA 2 database. (G) Detection of MMP2 expression in LUAD patients using TCGA dataset.


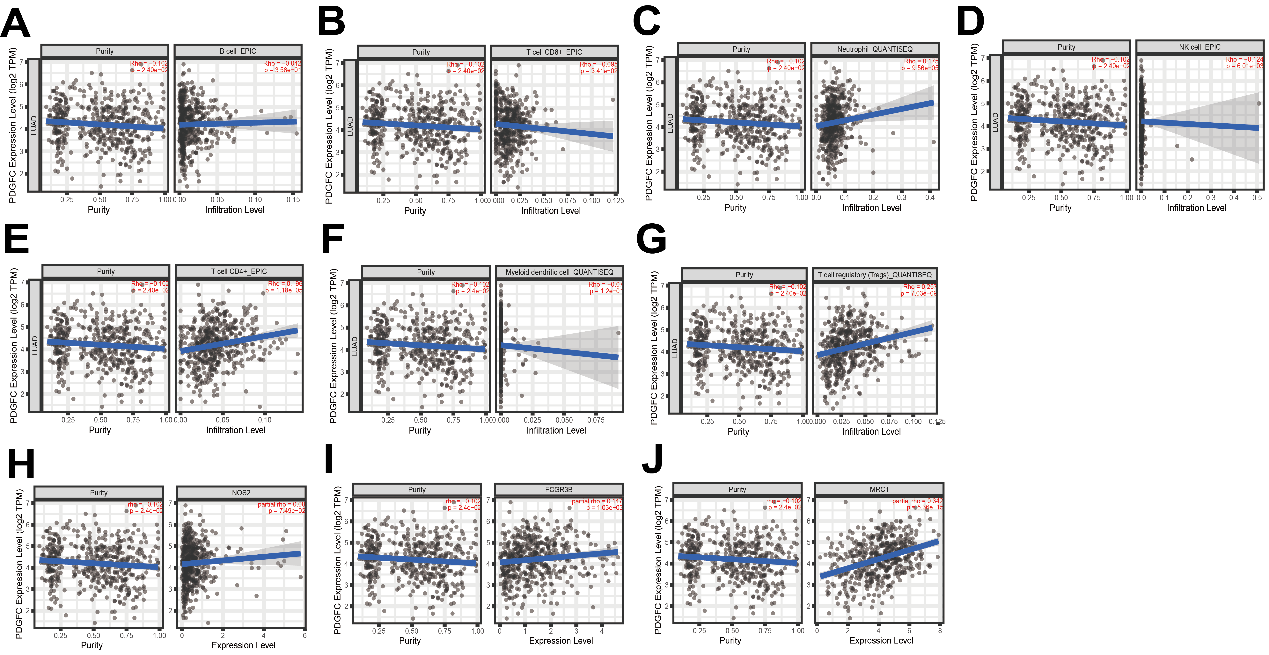


**Supplementary Figure S3. The effect of PDGFC on immune cell infiltration in LUAD** (A−G) TIMER 2.0 database analysis of the effect of PDGFC on immune cell infiltration in LUAD. (H−J) The correlation of PDGFC expression with N1 [iNOS (NOS2) and CD16b (FCGR3B)] N2 [CD206 (MCP1), and CD16b (FCGR3B)] markers in LUAD.


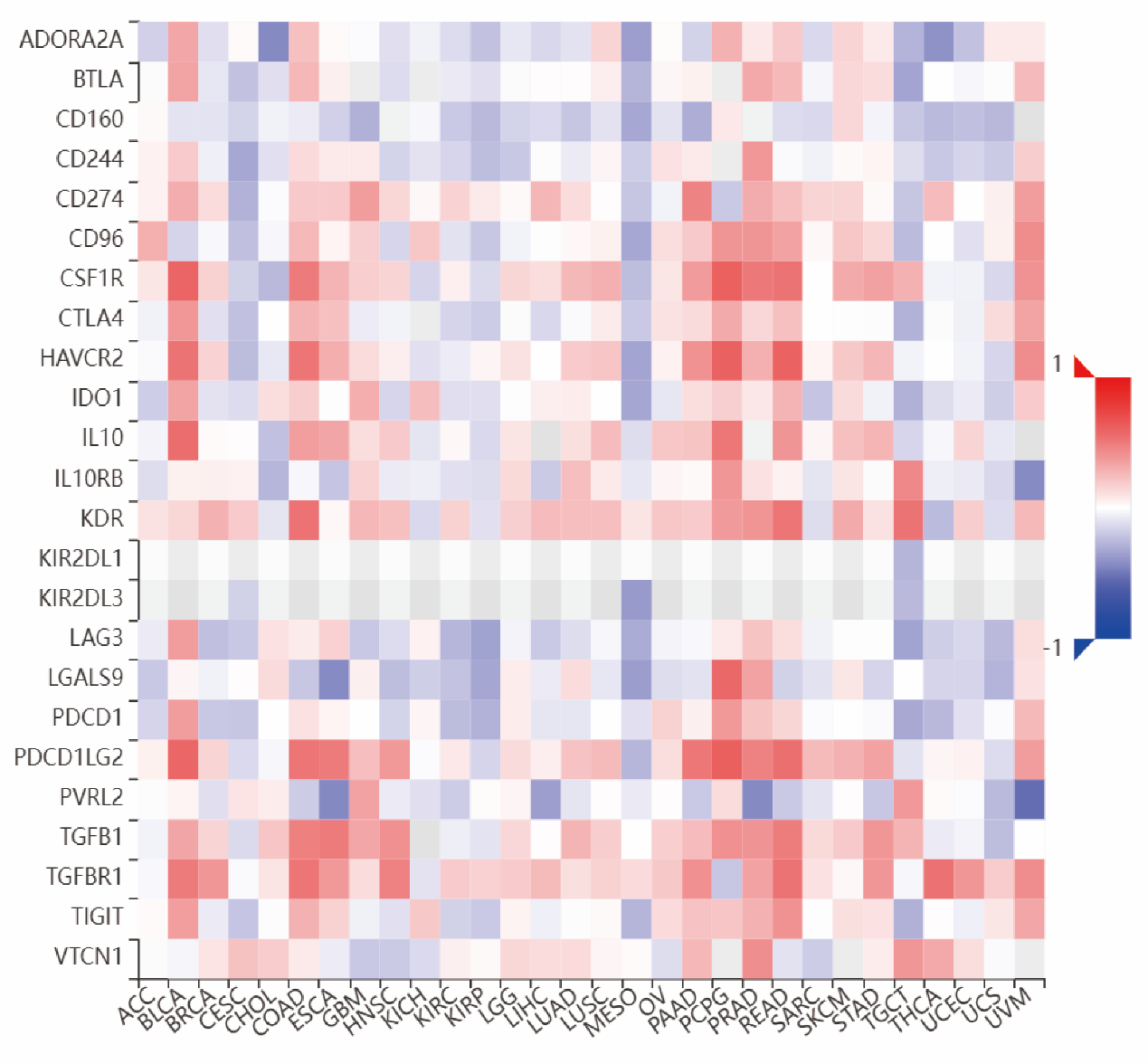


**Supplementary Figure S4.** **Spearman’s correlation of PDGFC with** **immunoinhibitors (TISIDB)** Relations between the immunoinhibitors and PDGFC expression.
